# Sulfonitric Treatment of Multiwalled Carbon Nanotubes and Their Dispersibility in Water

**DOI:** 10.3390/ma11122442

**Published:** 2018-12-02

**Authors:** Hui Liu, Jianfeng Wang, Jiachen Wang, Suping Cui

**Affiliations:** College of Materials Science and Engineering, Beijing University of Technology, Beijing 100124, China; liuhui9516@126.com (H.L.); wangjianfeng@bjut.edu.cn (J.W.); wjc9423@gmail.com (J.W.)

**Keywords:** carbon nanotubes, sulfonitric treatment, dispersion, hydrodynamic size

## Abstract

In this study, Multiwalled carbon nanotubes (MWCNTs) were oxidized by a mixture of sulfuric acid and nitric acid (*V*:*V* = 3:1) at 70 °C for 1, 2, and 4 h, respectively. The oxidized MWCNTs were characterized by N_2_ adsorption, Fourier-transform infrared spectroscopy (FT-IR), X-ray photoelectron spectroscopy (XPS), thermal gravimetric analysis (TGA), and Raman spectroscopy to determine the oxidation degree. The dispersion of the MWCNTs was investigated by UV-vis-NIR, SEM, and dynamic light scattering measurements. Results show that sulfonitric treatment increased the surface area and total pore volume and reduced the average pore diameter of MWCNTs. The treatment promoted the formation of oxidized species on the surface MWCNTs, as identified by FT-IR, TGA, and X-ray photoelectron spectroscopy measurements, and more oxygen-containing functional groups were generated when treatment time was extended. Moreover, a general relationship between oxidation degree and dispersibility of MWCNTs in water was established. UV-vis-NIR and dynamic light scattering measurements and SEM images revealed that MWCNTs with higher oxidation degree showed better dispersibility in water.

## 1. Introduction

Multiwalled carbon nanotubes (MWCNTs) have attracted a great deal of attention from research institutes and enterprises due to their excellent mechanical, electrical, thermodynamic, and chemical stability properties [1,2,3]. MWCNTs have been successfully used to prepare cementitious nanocomposites, energy storage materials, microelectronics, etc. [4,5] However, MWCNTs tend to agglomerate into bundles due to their large specific surface area and strong van der Waals forces, which would inhibit the nanoenhancement or modification effect [6,7]. Therefore, the homogeneous dispersion of carbon nanotubes has become a major challenge to fabricate MWCNT-composited cementitious materials. Many solvents and surfactants, such as liquid sodium metal ammonia solution methylcellulose [8], Pluronic F-127 and sodium dodecylbenzenesulfonate (SDBS) TritonX-100, and gum Arabic [9,10], have been employed to disperse MWCNTs. However, these additives may be not effective to disperse MWCNTs in cement pastes due to the possibility of preventing hydration, air entertainment, or undergoing reactions with other admixtures.

Oxidation treatment is an effective covalent functionalization approach to improve the physicochemical properties of carbon materials such as carbon fiber, activated carbon, and carbon nanotubes. Oxidization technology of carbon materials with sodium dichlororisocyanurate [11], nitric acid [12,13], or hydrogen peroxide [14] has been developed. Oxidation with nitric acid was shown to be more effective than hydrogen peroxide in functionalizing the carbon surface. Oxidized carbon materials are more efficient when used in ammonia adsorption [11], amide functionalization [15], polymerization catalysis [14], etc. Oxidized carbon nanotubes can be used directly to prepare nanocomposited materials or as a first step for surface modification [16,17,18]. The dispersive effect is attributed to two aspects: one is that oxidation improves the hydrophilicity of carbon nanotubes; another is that oxygen-containing functional groups, such as carboxyl produced by oxidation, play a role in electrostatic repulsion.

From the results presented in many studies, it can be summarized that the main factors affecting the oxidation processes of carbon nanotubes include oxidant types, temperature, and treatment time [19,20,21]. Oxidants containing concentrated nitric acid (HNO_3_) and sulfuric acid (H_2_SO_4_), potassium permanganate (KMnO_4_), or hydrogen peroxide (H_2_O_2_) were employed to modify carbon nanotubes [22,23,24,25]. A mixture of these oxidants, such as HNO_3_/H_2_SO_4_, H_2_O_2_/H_2_SO_4_ [26,27,28], HNO_3_/H_2_SO_4_/H_2_O_2_ [29], or H_2_SO_4_/HNO_3_/KMnO_4_ [30], can also achieve the oxidation of carbon nanotubes (CNTs), among which the HNO_3_/H_2_SO_4_ mixture is the most commonly used oxidant.

Chiu et al. [31] utilized a concentrated H_2_SO_4_/HNO_3_ mixture (*V*:*V* = 3:1) at 50 °C to treat MWCNTs and found that carboxyl groups (–COOH) and other functional groups (–OH, –C=O) formed on the surface of MWCNTs. The carboxylic groups can be observed at initial defect sites and newly created defect sites along the walls [32], which suggest that oxidization may lead to structural damages of MWCNTs. Su et al. [33] and Rosca et al. [34] also observed excessive oxidization and dissolution of MWCNTs when the treatment time was extended. Zhou et al. [35] reported that the appropriate acid treatment time would result in an effective number of nanodefects via suitable carboxyl functionalization, thus improving the dispersibility of MWCNTs. However, many groove defects appeared on the surface of MWCNTs treated with H_2_SO_4_ and HNO_3_ solution. Xing et al. [36] developed a sonochemical method to promote the density of functional groups (–C–O–, –C=O, and–COOH) and minimize structural damage to CNTs. Excessive oxidation of carbon nanotubes leads to the destruction of their structures, while the insufficient oxidation does not guarantee the formation of sufficient oxygen-containing functional groups to cause water-based dispersion. On the other hand, few studies have focused on the relation between the oxidation degree and the dispersion of MWCNTs.

For these considerations, a mixture of H_2_SO_4_ and HNO_3_ (*V*:*V* = 3:1) at 70 °C was considered to treat MWCNTs for 1–4 h. The content and type of functional groups formed on the surface of MWCNTs were investigated to identify the changes in composition and structural variations with treatment time. Finally, the relationship between the oxidation degree and the dispersion of MWCNTs was established. The definition of oxidation degree is a fractional conversion of C=C in the MWCNTs structure to oxygen-containing groups.

## 2. Materials and Methods

### 2.1. Materials

Multiwalled carbon nanotubes (CNT-1) were purchased from Beijing DK nano technology Co., Ltd. (Beijing, China). The physical properties of the carbon nanotubes are presented in Table 1. The oxidant consists of concentrated nitric acid (65%–68%) and concentrated sulfuric acid (95%–98%), provided by Beijing chemical works.

### 2.2. Characterization of MWCNT Powders

**Figure 1:**
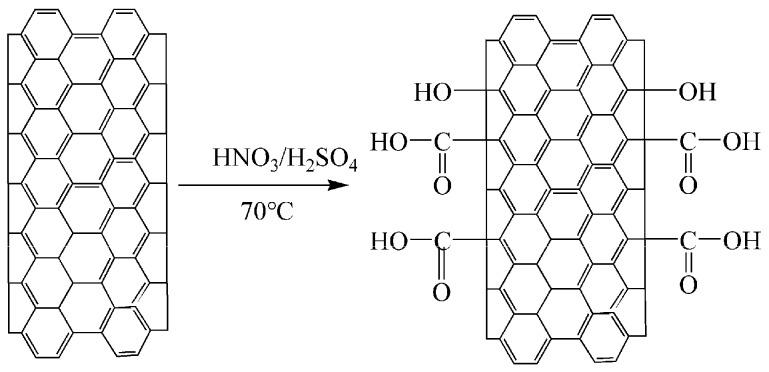
Reaction scheme for the acid treatment of MWCNTs.

For acid treatment, the surface modification of CNT-1 was performed with 1:3 (*V*:*V*) mixtures of concentrated HNO_3_ and H_2_SO_4_ at 70 °C for 1, 2, and 4 h. The reaction scheme for the oxidation is shown in Figure 1. The concentration of CNTs was fixed at 10 mg/ml. The functionalized CNTs were washed with water and ethanol to remove HNO_3_ and H_2_SO_4_ up to pH ~7 after cooling to 25 °C. Finally, the black solid was dried at 100 °C for 24 h. The samples treated for 1, 2, and 4 h were designated as CNT-2, CNT-3, and CNT-4, respectively.

The pore structures of pristine and oxidized MWCNTs were characterized by N_2_ adsorption using a surface area and porosimetry analyzer (Tristar TM II 3020, Micromeritics, Norcross, GA, USA). Oxygen-containing functional groups in MWCNTs were identified using Fourier-transform infrared spectroscopy (TENSOR 27, BRUKER, Bremen, Germany) in the wavenumber ranges of 1000–4000 cm^−1^. The samples were well mixed with KBr and pressed into a disk before being scanned by a Fourier-transform infrared spectrometer. X-ray photoelectron spectroscopy (XPS) (ESCALAB 250 XI, Thermo Scientific, Waltham, MA, USA) was performed to characterize the surface of MWCNTs. The content of C and O atoms can be obtained from the spectra recorded in the kinetic energy range of 0–1200 eV to quantitatively identify the relationship between oxidation degree and oxidation time. Thermogravimetry (TG) was performed by using NETZSCH STA-449-F3 (NETZSCH, Selb, Germany) from 30 to 900 °C under N_2_ atmosphere with a heating rate of 10 °C/min. The quantitative relationship between the treatment time and the content of oxidized species was established from the XPS and thermal gravimetric analysis (TGA) measurements. High-resolution Raman spectroscopy (LabRAM HR Evolution, HORIBA, Kyoto, Japan) was used to investigate the structural changes of the functionalized CNTs and the light source used was 532 nm.

### 2.3. Characterization of Aqueous MWCNT Dispersions

Dispersion of MWCNTs was analyzed by UV-vis-NIR spectroscopy (UV-4800, Shimadzu, Kyoto, Japan). The wavelength of spectrophotometer ranged from 200 to 800 nm for all measurements. As dispersed MWCNTs can absorb light in the UV-vis region effectively, the measured absorbance at a specific wavelength range could reflect the dispersive degree of primary or treated MWCNTs [37,38].

At first, samples were prepared from 10 mg of MWCNTs and 40 mL of water; then, the suspension was sonicated for 5 min at room temperature using a 200 W cup-horn high-intensity ultrasonic processor. After that, the suspensions were diluted up to 0.040–0.100 mg/mL followed by sonicating another 5 min before UV-vis-NIR measurement.

The particle size distribution (PSD) of MWCNT suspensions at a concentration of 0.040 mg/mL was characterized using a Malvern Zetasizer nano series instrument (Malvern Panalytical Ltd., Malvern, UK). The hydrodynamic particle size and particle size distribution can be obtained from the PSD measurement. The morphology of MWCNTs was taken without a carbon or gold film using an FEI QUANTA 250G (Field Electron and Ion Company, Hillsboro, OR, USA) in the low-vacuum mode of 70 Pa.

## 3. Results and Discussion

### 3.1. Nitrogen Adsorption Analyses

In order to investigate the pore structure of MWCNT samples, nitrogen adsorption isotherms were measured and analyzed. Table 2 shows the results of nitrogen adsorption analysis for the pristine and acidized MWCNTs. From the results, it was found that the surface area, average pore diameter, and total pore volume of pristine MWCNTs (CNT-1) were 80.72 m^2^/g, 16.14 nm, and 0.32 cm^3^/g, respectively. Obviously, acidification increased the surface area and total pore volume and reduced the average pore diameter. The effect was more remarkable when prolonging the treatment time. This phenomenon may be attributed to the destruction of the agglomeration structure resulting from sulfonitric treatment. 

### 3.2. FT-IR Spectroscopy

**Figure 2:**
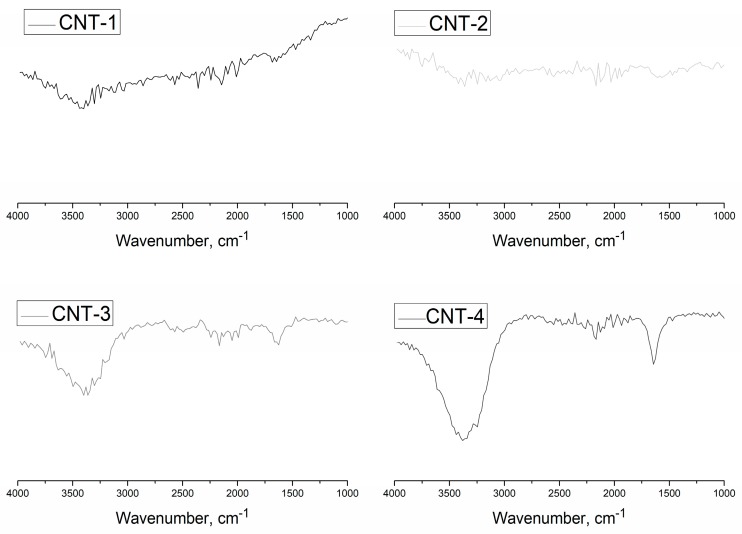
Fourier-transform infrared spectroscopy (FT-IR) spectra of MWCNTs after oxidation.

**Figure 3:**
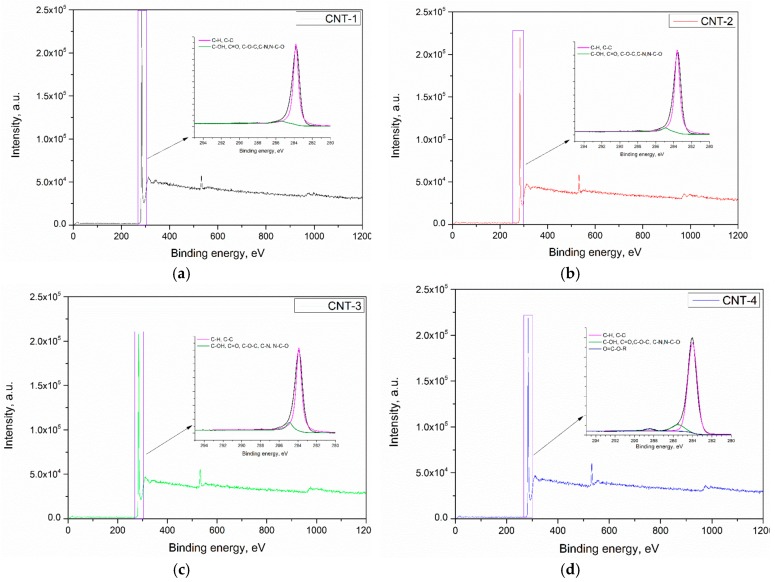
X-ray photoelectron spectroscopy (XPS) pattern of MWCNTs after treatment with acid: (**a**) CNT-1, (**b**) CNT-2, (**c**) CNT-3, and (**d**) CNT-4.

The FT-IR spectra of MWCNTs oxidized for 1, 2, and 4 h are shown in Figure 2. Results show that no obvious vibration peak presented in the FT-IR spectra of the pristine MWCNTs (CNT-1), which indicates that the primary MWCNTs contained few functional groups. Two obvious vibration peaks appeared in the FT-IR spectra of MWCNTs after sulfonitric treatment for 2 and 4 h. The stretching vibrations of –OH bonds were evidenced by the appearance of the band at 3420 cm^−1^, and the presence of bands near 1639 cm^−1^ were due to the –C=O stretching vibrations of –COOH and N-atoms that were covalently bonded to the carbon network (C=N). Figure 3 shows that the hydrophilic groups (–COOH, –OH) were generated on the surfaces of the MWCNTs, as observed by Zhang et al. [32]. However, N-containing groups, such as C–N or C–O–N observed in [39,40], were not detected through FT-IR measurement. These hydrophilic groups in MWCNTs may determine the dispersibility behavior in aqueous solutions. In addition, the above observations suggest that the oxidation degree increased with extended treatment time.

Although FT-IR analysis confirmed the functionalization of MWCNTs qualitatively, more details of MWCNTs resulting from sulfonitric treatment need to be further discussed.

### 3.3. XPS Characterization

**Figure 4:**
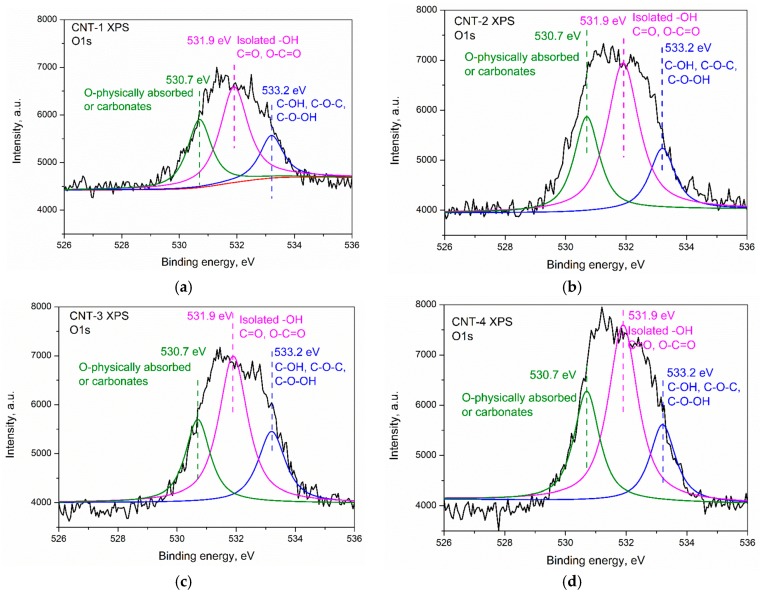
Deconvolution of the XPS O 1s peaks of the MWCNTs: (**a**) CNT-1, (**b**) CNT-2, (**c**) CNT-3, and (**d**) CNT-4.

Figure 3a–d presents the XPS spectrum of CNT-1 treated for 1, 2, and 4 h, respectively. Results show that the main components of the carbon nanotubes were C, O, and a small amount of Si. A characteristic peak for the C 1s located at a binding energy of 284.4 eV can be observed in the spectrum of primary and oxidized MWCNTs. The deconvolution of C 1s spectra of CNTs revealed the presence of C–H and C–C in carbon nanotubes, as confirmed by a main peak at ~284.1 eV. After sulfonitric treatment, functional groups, such as C–OH, C=O, C–N, C–O–C, and N–C–O, were generated. In addition, O=C–O–R was generated in the CNTs after being oxidized for 4 h. The XPS O 1s peak appeared at a binding energy of 531 eV, which confirmed that some oxidized species were generated on the surface of treated MWCNTs. Deconvolution of the O 1s peak of pristine and oxidized MWCNTs are presented in Figure 4a–d. Three peaks appeared at 533.2, 531.9, and 530.7 eV, which confirmed the generation of carboxylic, hydroxyl species, and physically adsorbed oxygen/carbonates, as reported by Datsyuk [31]. The quantification of possible band assignments determined from XPS O 1s deconvolution are presented in Table 3. Results show that the low content of the carboxylic and hydroxyl functions (1.10% and 0.32%) presented on the surface of MWCNTs. The content of such functions increased from extending the oxidation time and reached 3.03% and 1.21%, respectively.

The atomic percentages of oxygen, carbon, and silicon on the pristine/acidized MWCNTs characterized by XPS measurements are summarized in Table 4. There is a remarkable increase in oxygen content of MWCNTs after being subjected to sulfonitric treatment. When oxidized by an HNO_3_/H_2_SO_4_ mixture, MWCNTs underwent some oxidative cleavage of C–C bonds, converting sp^2^ hybridized carbon atoms into oxidized sp^3^ ones to form oxygen-containing functionalized groups such as carboxylic and hydroxyl. Therefore, the overall amount of oxygen increased with treatment time and the O/C ratio in the sample also increased significantly from 2.11% to 6.13% (Table 4) after treatment for 4 h, which manifested oxidized species generated on the surface of MWCNTs during the sulfonitric treatment process. As expected, the oxidation degree of MWCNTs increased with prolonged treatment time.

### 3.4. Thermogravimetric Analysis

**Figure 5:**
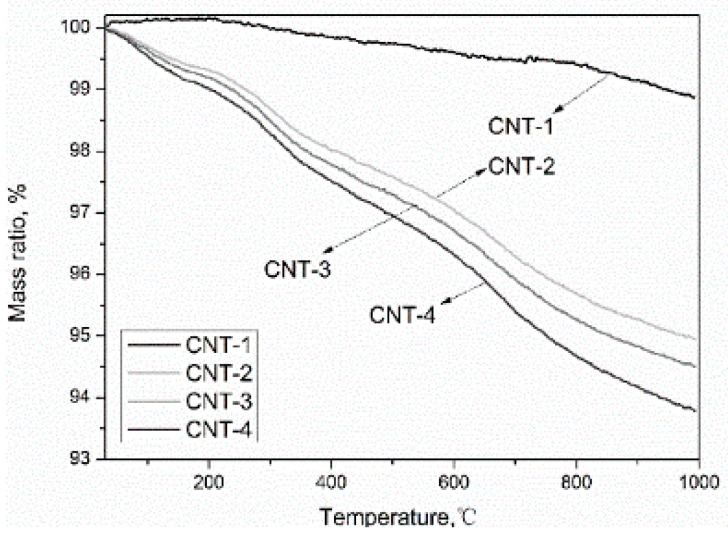
Thermal gravimetric analysis (TGA) curve of acid-treated MWCNTs.

Figure 5 shows the TGA curves measured in the temperature range from 30 to 900 °C with a heating rate of 10 °C/min in the N_2_ atmosphere before and after oxidation of MWCNTs. The weight loss process can be divided into four stages. The first stage occurs below 150 °C, which is attributed to the loss of moisture [28,41]. The second weight loss stage from 150 to 350 °C is ascribed to the decarboxylation of –COOH presented on the MWCNTs [42]. The third step in the range between 350 and 500 °C is due to the elimination of –OH [43]. The last one occurs between 500 and 900 °C and is attributed to the thermal oxidation of the remaining amorphous carbon [39]. The mass loss ratio calculated from the curve at different temperature ranges is presented in Table 5. The content of the carboxyl and hydroxyl groups increased with the extension of oxidation time, which is in agreement with the results obtained from FT-IR and XPS analyses. Among the four samples, the pristine MWCNTs (CNT-1) exhibited the lowest total mass loss (1.22%) up to 900 °C, which conformed to the results obtained by Chiu et al. [31], due to the good thermal stability of the MWCNTs with a perfect hexagon crystal structure. This means oxidation decreased the thermal stability of MWCNTs. The worsening thermal stability of MWCNTs as a result of the oxidation is not detrimental for the application of the formulated materials when used in cementitious materials, as the service temperature of cement-based materials is generally not more than 100 °C.

### 3.5. Raman Spectroscopy

**Figure 6:**
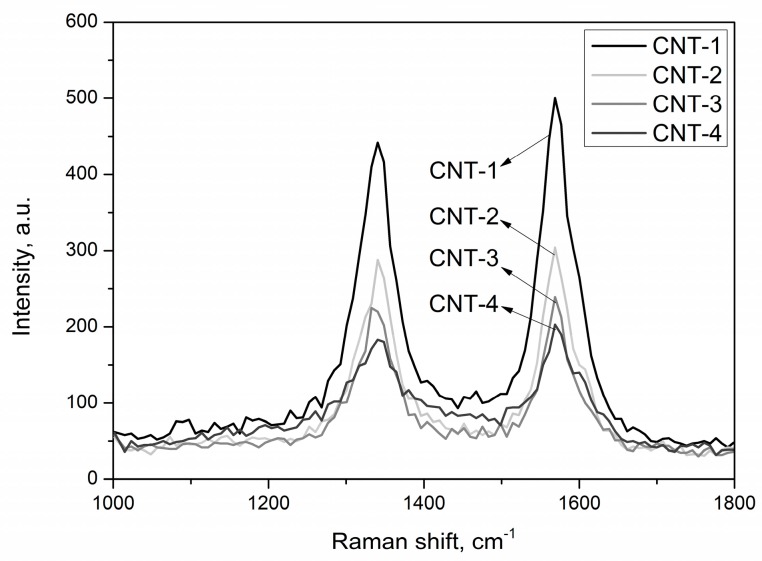
Raman spectra of MWCNTs after treatment with acid.

In order to identify the structural integrity of carbon nanotubes during the treatment process, Raman spectroscopy was used due to its high sensitivity to the structural change of MWCNTs. The Raman spectra of pristine and treated MWCNTs with acid for 1, 2, and 4 h are presented in Figure 6. Results show that the main feature of each spectra was the two characteristic bands: the G-band (graphic band) at 1570 cm^−1^ accounted for the degree of crystallinity of graphitic sheets, and the D-band (disorder band) at 1338 cm^−1^ represented the crystal defect degree of graphitic sheets [44]. 

The intensity ratio of the D and G bands (I_D_/I_G_) was employed to determine the degree of structural defects or disorders in MWCNTs [45,46]. The position of the peak D and G (I_D_/I_G_) ratio is presented in Table 6. From the results presented in Figure 6 and Table 6, it can be seen that the value of I_D_/I_G_ increased with increasing treatment time, which suggests that more defects formed in the surface of MWCNTs with longer treatment time. Similar results were also found by Chiu et al. [31] through chemical modification of MWCNTs. 

From the Raman spectra of MWCNTs, an upfield shift of the D-band for the MWCNTs with sulfonitric treatment was noticed, which may correlate with the intertubular coupling resulting from repulsive van der Waals forces [47]. 

The above results were used to qualitatively and quantitatively analyze the effect of sulfonitric treatment on the composition and structure of MWCNTs. In summary, the sulfonitric treatment of MWCNTs resulted in decreasing the crystallinity of MWCNTs with the generation of oxygen-containing groups such as –OH and –COOH. The effect was more evident when increasing the oxidation time. To establish the relationship between the degree of oxidation and the dispersion of MWCNTs, the suspensions were characterized by UV-vis-NIR and dynamic light scattering measurements.

### 3.6. The Dispersion of Sulfonitric Treated MWCNTs

#### 3.6.1. The Relationship between the Concentration of MWCNTS and Absorbance

**Figure 7:**
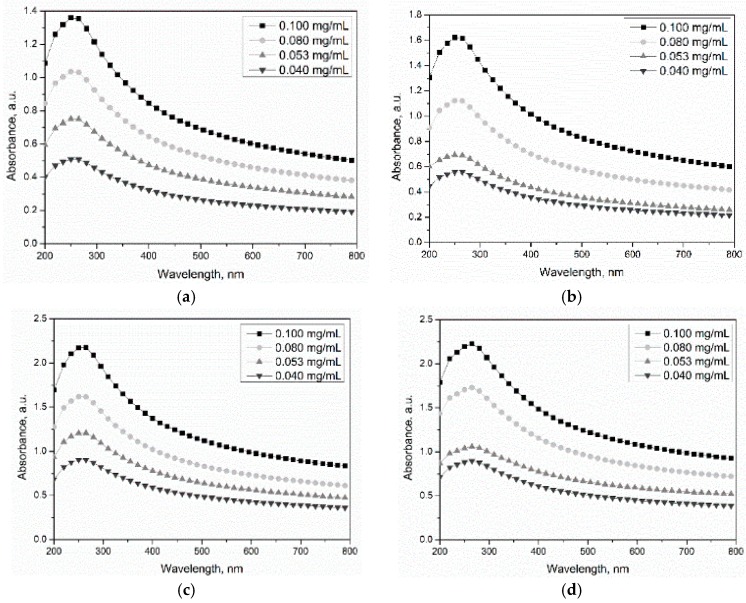
UV-vis-NIR spectra of oxidized MWCNT suspensions: (**a**) CNT-1, (**b**) CNT-2, (**c**) CNT-3, and (**d**) CNT-4.

The UV-vis-NIR spectrum was used to characterize the dispersion of MWCNTs in water due to individual nanotubes having strong absorption in the UV-vis region, while bundled CNTs are not active. Figure 7a–d depicts the absorbance spectra of UV-vis-NIR scans between 200 and 800 nm for MWCNTs at different concentrations (0.040–0.100 mg/mL). The relationships between the absorbance at 265, 350, 500, and 700 nm and concentrations are also presented in Figure 7. From the results, the maximum absorption for the MWCNTs was found at wavelength of 265 nm, and no position shift was observed for the four MWCNTs. In addition, the absorption value of MWCNTs increased linearly with the concentration of carbon nanotubes in the suspensions (R^2^ ≥ 95%), which is in accordance with the Lambert-Beer law. Figure 7 shows that the fitted line obtained from the absorbance at 265 nm showed the maximum slope, which implies that the absorbance at 265 nm was more sensitive to the concentration of MWCNTs in suspensions. According to the Lambert-Beer law, the absorbance value (A) of MWCNT suspensions obtained from UV-vis-NIR spectroscopy measurement can be expressed as:

A = lg (1/T) = lg (I_0_/I) = Kbc (1)

T: transmission;

I_0_ and I: intensity of the measuring beam before/after passing through the sample;

K: molar absorption coefficient of MWCNTs;

c: concentration of MWCNTs;

b: path length of the measuring beam in the sample.

For the same MWCNT type, the absorbance value is proportional to the concentration of MWCNTs in suspensions due to the same value of K and b. However, the distinguished slopes of MWCNTs treated for different time can be observed in Figure 7 and are related to the oxidization degree of MWCNTs. 

#### 3.6.2. The Relationship between the Oxidation Degree and the Absorbance

**Figure 8:**
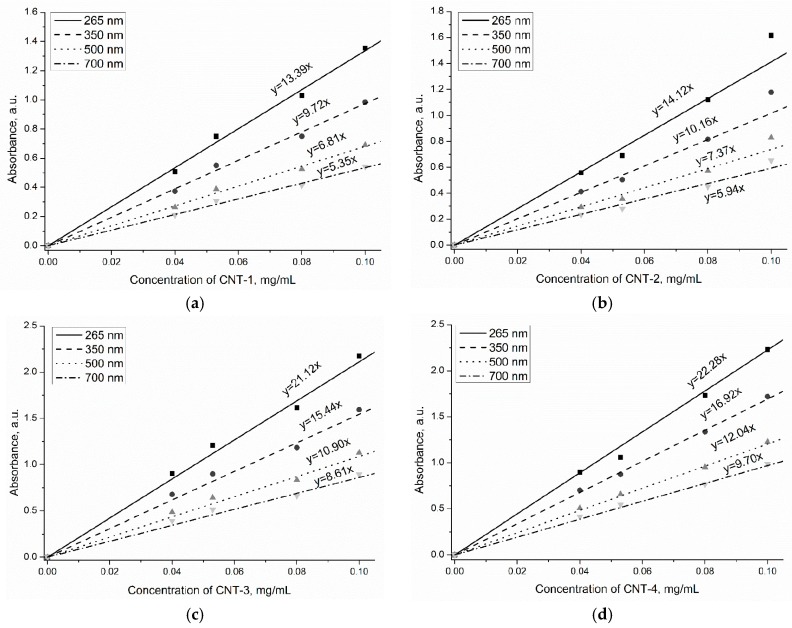
Calibration lines of MWCNTs after treatment: (**a**) CNT-1, (**b**) CNT-2, (**c**) CNT-3, and (**d**) CNT-4.

The primary CNTs and sulfonitric-treated CNTs for 1, 2, and 4 h were dispersed in water at different concentrations (0.040–0.100 mg/mL). Figure 8a–d also shows the absorbance of MWCNT suspensions at 0.040, 0.053, 0.080, and 0.100 mg/mL, respectively. As expected, a clear tendency for the absorbance value of MWCNTs to increase with extended oxidation time can be noticed. Among the MWCNTs in this study, CNT-4 exhibited the optimal dispersibility at the same concentration. This is due to the hydrophobic groups providing repulsive forces among the carbon nanotubes, thus improving the dispersion of CNTs. For HNO_3_/H_2_SO_4_-treated MWCNTs, the distinguished absorbance value was detected at the same MWCNT concentration, which indicated that the K value varied with treatment time. The molar absorption coefficient of CNT-1–CNT-4 was assumed as K_1_, K_2_, K_3_, and K_4_. According to the Lambert-Beer law, the values of K_2_/K_1_, K_3_/K_1_ and K_4_/K_1_ can be calculated, which were about 1.31, 1.50, and 1.75, respectively. Therefore, the increase of the molar absorption coefficient resulting from oxidization may contribute to the positive effect on the dispersion of MWCNTs in water.

### 3.7. The PSD of MWCNT Suspensions

**Figure 9:**
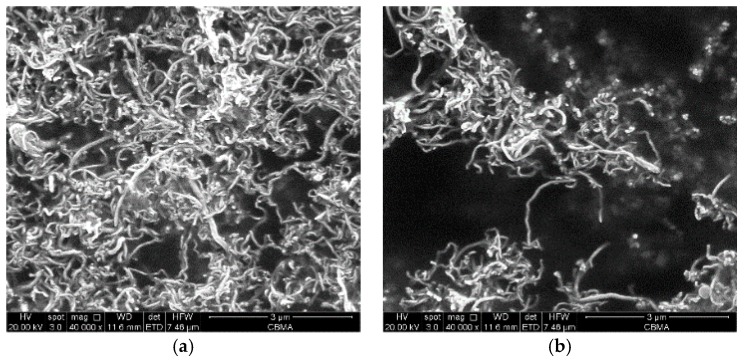
The images of the dispersed samples in water. (**a**) CNT-1; (**b**) CNT-4.

For a better understanding of the dispersive effect for MWCNTs after oxidation treatment, the intensity of particle size distribution was measured by dynamic light scattering. Figure 9 shows the images of the pristine/oxidized MWCNTs dispersed in water at a concentration of 0.040 mg/mL. The morphology of CNT-1 shows that the pristine MWCNTs agglomerated heavily into particles due to strong van der Waals forces. Notably, the MWCNTs after treatment (CNT-4) were better dispersed in water than pristine MWCNTs (CNT-1). This suggests oxidized MWCNTs exhibit better dispersibility in water. 

**Figure 10:**
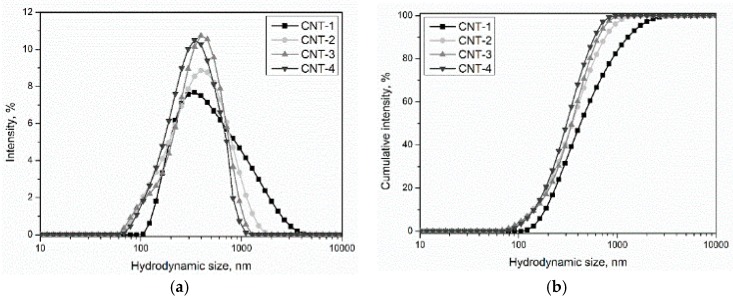
The hydrodynamic size distribution of MWCNT suspensions: (**a**) hydrodynamic size distribution and (**b**) cumulative intensity.

The hydrodynamic size distribution of MWCNTs at a concentration of 0.040 mg/mL is depicted in Figure 10 and the average hydrodynamic size of MWCNT suspensions is shown in Table 7. Experimental data indicated that MWCNT particle sizes in the suspensions ranged from 60 to 4000 nm, which means the primary and treated CNT suspensions were in large agglomerates or bundles. By comparing with the PSD of primary and treated MWCNTs, it is notable that oxidation treatment led to a dispersive effect on MWCNTs, as indicated by the decrease of hydrodynamic size from 609.9 to 364.3 nm. In addition, the hydrodynamic size seemed to decrease with prolonged treatment time. Among the modified MWCNTs, CNT-4 exhibited the best dispersibility, as indicated by the lowest hydrodynamic size. Combined with the results presented in Section 3.6, we can conclude that the sulfonitric treatment of MWCNTs using a HNO_3_/H_2_SO_4_ mixture had a positive effect on their dispersion in water, and the effect was even more remarkable with prolonged treatment time.

## 4. Conclusions

Sulfonitric treatment of MWCNTs and their dispersibility in water were investigated using various measurements. Nitrogen adsorption analyses showed that sulfonitric treatment increased the surface area and total pore volume and reduced the average pore diameter of MWCNTs. The treatment promoted the generation of oxygen-containing species such as carboxylic, hydroxyl species, and physically adsorbed oxygen/carbonates. The effects were more obvious when increasing oxidation degree by prolonging the treatment time, as confirmed by quantitative analysis of these species and Raman spectroscopy measurements. Oxidized MWCNTs exhibited a higher dispersion efficiency in water than pristine MWCNTs, indicated by an increasing UV absorbance value and a decreasing average hydrodynamic size. SEM observations also confirmed the dispersive effect of sulfonitric treatment on MWCNTs. Moreover, the absorption value of MWCNTs increased linearly with the concentration of carbon nanotubes in the suspensions. The oxidation degree of MWCNTs was proportional to their dispersibility in water, which may relate to the formation of oxygen-containing species and the molar absorption coefficient.

## Figures and Tables

**Table 1:** Properties of pristine multiwalled carbon nanotubes (MWCNTs) (CNT-1).

Type	Inside Diameter	Outside Diameter	Length	Density	Special Surface Area
CNT-1	3–10 nm	8–30 nm	10–50 μm	2.1 g/cm^3^	>110 cm^2^/g

**Table 2:** Results of nitrogen adsorption analysis for the carbon nanotube (CNT) samples.

Samples	BET Surface Area (m^2^/g)	Average Pore Diameter (4V/A) (nm)	Total Pore Volume (cm^3^/g)
CNT-1	80.72	16.14	0.32
CNT-2	113.22	15.09	0.43
CNT-3	118.48	14.50	0.44
CNT-4	130.80	14.07	0.51

**Table 3:** Assignments of the O1s spectra fitting into chemical groups for XPS spectra of pristine and functionalized CNTs.

Samples	Content, %		
	533.2 eV	531.9 eV	530.7 eV
	C–OH C–O–C C–O–OH	C=O O–C=O	O-Physically Absorbed or Carbonates
CNT-1	0.32	1.10	0.64
CNT-2	0.94	1.36	1.27
CNT-3	1.17	2.40	1.44
CNT-4	1.21	3.03	1.51

**Table 4:** Atomic content of MWCNTs after acid treatment.

Samples	Atomic, %			O/C, %
	C 1s	O 1s	Si 2p	
CNT-1	97.61	2.06	0.33	2.11
CNT-2	96.12	3.57	0.31	3.71
CNT-3	94.66	5.01	0.33	5.29
CNT-4	93.84	5.75	0.41	6.13

**Table 5:** The mass loss of MWCNTs after treatment with acid obtained from the TGA curve.

Sample	Mass Loss, %			Total Mass Loss, %
	150–350 °C	350–500 °C	500–900 °C	
CNT-1	0.08	0.18	0.86	1.22
CNT-2	1.19	0.71	2.63	5.06
CNT-3	1.32	0.87	2.78	5.50
CNT-4	1.41	0.75	3.16	6.22

**Table 6:** I_D_/I_G_ ratio of MWCNTs after acid treatment.

Sample	Position of Peak D and G	I_D_/I_G_
CNT-1	(1335, 1570)	0.847
CNT-2	(1342, 1569)	0.936
CNT-3	(1339, 1570)	0.958
CNT-4	(1337, 1569)	1.050

**Table 7:** The average hydrodynamic size of MWCNT suspensions.

Sample	Average Hydrodynamic Size, nm
CNT-1	609.9
CNT-2	437.2
CNT-3	402.4
CNT-4	364.3

## References

[1] Siddique R., Mehta A. (2014). Effect of carbon nanotubes on properties of cement mortars. Constr. Build. Mater..

[2] Yu M.F., Lourie O., Dyer M.J., Moloni K., Kelly T.F., Ruoff R.S. (2000). Strength and breaking mechanism of multiwalled carbon nanotubes under tensile load. Science.

[3] Walters D.A., Ericson L.M., Casavant M.J., Liu J. (1999). Elastic strain of freely suspended single-wall carbon nanotube ropes. Appl. Phys. Lett..

[4] Hanus M.J., Harris A.T. (2013). Nanotechnology innovations for the construction industry. Prog. Mater. Sci..

[5] Tian M., Wang W., Liu Y., Jungjohann K.L., Harris C.T., Lee Y.C., Yang R. (2015). A three-dimensional carbon nano-network for high performance lithium ion batteries. Nano Energy.

[6] Liu Y., Yu L., Zhang S., Yuan J., Shi L., Zheng L. (2010). Dispersion of multiwalled carbon nanotubes by ionic liquid-type gemini imidazolium surfactants in aqueous solution. Coll. Surf. A.

[7] Ma P.C., Siddiqui N.A., Marom G., Kim J.K. (2010). Dispersion and functionalization of carbon nanotubes for polymer-based nanocomposites: A review. Comp. Part A.

[8] Fogden S., Howard C.A., Heenan R.K., Skipper N.T., Shaffer M.S. (2012). Scalable method for the reductive dissolution, purification, and separation of single-walled carbon nanotubes. ACS Nano.

[9] Parveen S., Rana S., Fangueiro R., Paiva M.C. (2015). Microstructure and mechanical properties of carbon nanotube reinforced cementitious composites developed using a novel dispersion technique. Cem. Concr. Res..

[10] Han B., Sun S., Ding S., Zhang L., Yu X., Ou J. (2015). Review of nanocarbon-engineered multifunctional cementitious composites. Compos. A Appl. Sci. Manuf..

[11] Molina-Sabio M., Gonçalves M., Rodríguez-Reinoso F. (2011). Oxidation of activated carbon with aqueous solution of sodium dichloroisocyanurate: Effect on ammonia adsorption. Microporous Mesoporous Mater..

[12] Bleda-Martínez M.J., Lozano-Castelló D., Morallón E., Cazorla-Amorósa D., Linares-Solano A. (2006). Chemical and electrochemical characterization of porous carbon materials. Carbon.

[13] Zubizarreta L., Menéndez J.A., Job N., Marco-Lozar J.P., Pirard J.P., Pis J.J., Linares-Solano A., Cazorla-Amorós D., Arenillas A. (2010). Ni-doped carbon xerogels for H_2_ storage. Carbon.

[14] Barrientos-Ramírez S., Montes de Oca-Ramírez G., Ramos-Fernández E.V., Sepúlveda-Escribano A., Pastor-Blas M.M., González-Montiel A., Rodríguez-Reinoso F. (2011). Influence of the surface chemistry of activated carbons on the ATRP catalysis of methyl methacrylate polymerization. Appl. Catal. A.

[15] Mostazo-López M.J., Ruiz-Rosas R., Morallón E., Cazorla-Amorósa D. (2015). Generation of nitrogen functionalities on activated carbons by amidation reactions and Hofmann rearrangement: Chemical and electrochemical characterization. Carbon.

[16] Kim S.D., Park S.J., Lee Y.K. (2008). Chemical surface treatment for highly improved dispersibility of multi-walled carbon nanotubes in water. J. Dispers. Sci. Technol..

[17] Balasubramanian K., Burghard M. (2005). Chemically functionalized carbon nanotubes. Small.

[18] Gojny F.H., Nastalczyk J., Roslaniec Z., Schulte K. (2003). Surface modified multi-walled carbon nanotubes in CNT/epoxy-composites. Chem. Phys. Lett..

[19] Karousis N., Tagmatarchis N., Tasis D. (2010). Current progress on the chemical modification of carbon nanotubes. Chem. Rev..

[20] Hui H., Zhao B., Itkis M.E., Haddon R.C. (2004). Nitric acid purification of single-walled carbon nanotubes. J. Phys. Chem. B.

[21] MartíNez M.T., Calleja M.A., Benito A.M., Cochet M., Seeger T., Ansón A., Schreiber J., Gordon C., Marhic C., Chauvet O. (2003). Sensitivity of single wall carbon nanotubes to oxidative processing: Structural modification, intercalation and functionalization. Carbon.

[22] Kukovecz A., Kramberger Ch., Holzinger M., Kuzmany H., Schalko J., Mannsberger M., Hirsch A. (2015). On the stacking behavior of functionalized single-wall carbon nanotubes. J. Phys. Chem. B.

[23] Liu L., Qin Y., Guo Z.X., Zhu D. (2003). Reduction of solubilized multi-walled carbon nanotubes. Carbon.

[24] Hayashi S., Handa S., Tsubokawa N. (2015). Introduction of peroxide groups onto carbon black surface by radical trapping and radical graft polymerization of vinyl monomers initiated by the surface peroxide groups. J. Polym. Sci. A Polym. Chem..

[25] Marega R., Accorsi G., Meneghetti M., Parisini A., Prato M., Bonifazi D. (2009). Cap removal and shortening of double-walled and very-thin multi-walled carbon nanotubes under mild oxidative conditions. Carbon.

[26] Kuznetsova A., Popova I., Yates J.T., Bronikowski M.J., Huffman C.B., Liu J., Smalley R.E., Hwu H., Chen J. (2001). Oxygen-containing functional groups on single-wall carbon nanotubes:  NEXAFS and vibrational spectroscopic studies. J. Am. Chem. Soc..

[27] Ziegler K.J., Gu Z., Peng H., Flor E.L., Hauge R.H., Smalley R.E. (2005). Controlled oxidative cutting of single-walled carbon nanotubes. J. Am. Chem. Soc..

[28] Datsyuk V., Kalyva M., Papagelis K., Parthenios J., Tasis D., Siokou A., Kallitsisa I., Galiotisa C. (2008). Chemical oxidation of multiwalled carbon nanotubes. Carbon.

[29] Avilés F., Cauich-Rodríguez J.V., Moo-Tah L., May-Pat A., Vargas-Coronado R. (2009). Evaluation of mild acid oxidation treatments for MWCNT functionalization. Carbon.

[30] Hiura H., Ebbesen T.W., Tanigaki K. (2010). Opening and purification of carbon nanotubes in high yields. Adv. Mater..

[31] Chiu W.M., Chang Y.A. (2008). Chemical modification of multiwalled carbon nanotube with the liquid phase method. J. Appl. Polym. Sci..

[32] Zhang J., Zou H.L., Qing Q., Yang Y., Li Q., Liu Z., Guo X., Du Z. (2015). Effect of Chemical Oxidation on the Structure of Single-Walled Carbon Nanotubes. J. Phys. Chem. B.

[33] Su S.H., Chiang W.T., Lin C., Yokoyama M. (2008). Multi-wall carbon nanotubes: Purification, morphology and field emission performance. Phys. E Low Dimens. Syst. Nanostruct..

[34] Rosca I.D., Watari F., Uo M., Akasaka T. (2005). Oxidation of multiwalled carbon nanotubes by nitric acid. Carbon.

[35] Zhou W., Sasaki S., Kawasaki A. (2014). Effective control of nanodefects in multiwalled carbon nanotubes by acid treatment. Carbon.

[36] Xing Y., Li L., Chusuei C., Hull R.V. (2005). Sonochemical oxidation of multiwalled carbon nanotubes. Langmuir.

[37] Grossiord N., Regev O., Loos J., Meuldijk J., Koning C.E. (2005). Time-dependent study of the exfoliation process of carbon nanotubes in aqueous dispersions by using UV-visible spectroscopy. Anal. Chem..

[38] Yu J., Grossiord N., Koning C.E., Loos J. (2007). Controlling the dispersion of multi-wall carbon nanotubes in aqueous surfactant solution. Carbon.

[39] Wang L., Liu N., Guo Z., Wu D., Chen W., Chang Z., Wang J. (2016). Nitric acid-treated carbon fibers with enhanced hydrophilicity for candida tropicalis immobilization in xylitol fermentation. Materials.

[40] Gómez S., Rendtorff N.M., Aglietti E.F., Sakka Y., Suarez G. (2017). Intensity of sulfonitric treatment on multiwall carbon nanotubes. Chem. Phys. Lett..

[41] Park O.K., Kim N.H., Yoo G.H., Rhee K.Y., Lee J.H. (2010). Effects of the surface treatment on the properties of polyaniline coated carbon nanotubes/epoxy composites. Compos. B Eng..

[42] Tang M., Dou H., Sun K. (2006). One-step synthesis of dextran-based stable nanoparticles assisted by self-assembly. Polymer.

[43] Grandi S., Magistris A., Mustarelli P., Quartarone E., Tomasi C., Meda L. (2006). Synthesis and characterization of SiO_2_–PEG hybrid materials. J. Non Cryst. Solids.

[44] Hou P., Liu C., Tong Y., Xu S., Liu M., Chen H. (2001). Purification of single-walled carbon nanotubes synthesized by the hydrogen arc-discharge method. J. Mater. Res..

[45] Flahaut E., Laurent C., Peigney A. (2005). Catalytic CVD synthesis of double and triple-walled carbon nanotubes by the control of the catalyst preparation. Carbon.

[46] Dresselhaus M.S., Dresselhaus G., Jorio A. (2007). Raman spectroscopy of carbon nanotubes in 1997 and 2007. J. Phys. Chem. C.

[47] Chen C., Ogino A., Wang X., Nagatsu M. (2001). Oxygen functionalization of multiwall carbon nanotubes by Ar/H_2_O plasma treatment. Diam. Relat. Mater..

